# Genome Analysis of Environmental and Clinical *P. aeruginosa* Isolates from Sequence Type-1146

**DOI:** 10.1371/journal.pone.0107754

**Published:** 2014-10-15

**Authors:** David Sánchez, Margarita Gomila, Antonio Bennasar, Jorge Lalucat, Elena García-Valdés

**Affiliations:** 1 Microbiologia, Departament de Biologia, Edifici Guillem Colom, Universitat de les Illes Balears, Campus UIB, Palma de Mallorca, Spain; 2 Instituto Universitario de Investigación en Ciencias de la Salud (IUNICS-UIB) Universitat de les Illes Balears, Campus UIB, Palma de Mallorca, Spain; 3 Institut Mediterrani d'Estudis Avançats (IMEDEA, CSIC-UIB), Campus UIB, Palma de Mallorca, Spain; The University of Hong Kong, Hong Kong

## Abstract

The genomes of *Pseudomonas aeruginosa* isolates of the new sequence type ST-1146, three environmental (P37, P47 and P49) and one clinical (SD9) isolates, with differences in their antibiotic susceptibility profiles have been sequenced and analysed. The genomes were mapped against *P. aeruginosa* PAO1-UW and UCBPP-PA14. The allelic profiles showed that the highest number of differences were in “Related to phage, transposon or plasmid” and “Secreted factors” categories. The clinical isolate showed a number of exclusive alleles greater than that for the environmental isolates. The phage Pf1 region in isolate SD9 accumulated the highest number of nucleotide substitutions. The ORF analysis of the four genomes assembled *de novo* indicated that the number of isolate-specific genes was higher in isolate SD9 (132 genes) than in isolates P37 (24 genes), P47 (16 genes) and P49 (21 genes). CRISPR elements were found in all isolates and SD9 showed differences in the spacer region. Genes related to bacteriophages F116 and H66 were found only in isolate SD9. Genome comparisons indicated that the isolates of ST-1146 are close related, and most genes implicated in pathogenicity are highly conserved, suggesting a genetic potential for infectivity in the environmental isolates similar to the clinical one. Phage-related genes are responsible of the main differences among the genomes of ST-1146 isolates. The role of bacteriophages has to be considered in the adaptation processes of isolates to the host and in microevolution studies.

## Introduction

*Pseudomonas aeruginosa* is a Gram negative, aerobic, rod-shaped, gammaproteobacterium with polar inserted flagella. Environmental isolates of this ubiquitous bacterium are highly versatile and adapt easily to a large variety of natural ecosystems, although water is considered to be the primary habitat of this microorganism [1], [2]. *P. aeruginosa* can cause a wide range of opportunistic infections in animals and humans [3]. The colonisation of this broad spectrum of habitats results from the ability to exploit many different nutrition sources and the high potential to adapt to new (or changing) environmental conditions [4].

The genomes of *P. aeruginosa* strains are larger than those of most sequenced bacteria, varying from 5.2 to 7.1 Mbp [5]. The divergence in genome size is caused by the so-called accessory genome. The core genome, with a few exceptions of loci that are subject to diversifying selection, is highly conserved among clonal complexes and shows sequence diversities of 0.5–0.7% [6]–[8]. The elements of the accessory genome have apparently been acquired by horizontal gene transfer from different sources, including other species or genera. Therefore, a *P. aeruginosa* chromosome is often described as a mosaic structure of a conserved core genome frequently interrupted by the inserted portions of the accessory genome. The individual mosaics also show remarkable plasticity [9], [10]. The ongoing acquisition of new foreign DNA, the mobilization of prophages, larger or smaller deletion events, mutations of single nucleotides and even chromosomal inversions [8], [11]–[15], are potentially affecting portions of the core and the accessory genome, and these processes continuously modify the genome and modulate the phenotype of a *P. aeruginosa* strain, thus differentiating the strains from each other.

*P. aeruginosa* strains have been preferentially studied in cystic fibrosis (CF) patients in the clinical context. Fewer environmental studies have been conducted than clinical studies [16]–[18]. The ability of *P. aeruginosa* to adapt to different habitats provides an excellent model for examining the mechanisms used by environmental strains of the ubiquitous *P. aeruginosa* species. The genomic structure of *P. aeruginosa* strains has also been analysed in detail predominantly in the clinical isolates of patients with CF. Klockgether et al. [10] have suggested that the sequencing of strains from environmental habitats should provide an unbiased overview of the genetic repertoire of the *P. aeruginosa* populations. Few environmental strains have been sequenced thus far [1], [19]–[21]. More than 166 *P. aeruginosa* genome sequences are available on the National Centre of Biotechnology Information (NCBI), and less than 10% are of environmental strains. In a screening of *P. aeruginosa* strains isolated from water and clinical specimens in Mallorca (Spain) a MultiLocus Sequence typing (MLST) analysis was performed, and a new sequence type ST-1146 was found. Interestingly this was the unique sequence type that included environmental and clinical strains. In the present study, 4 genomes of intimately related isolates from this sequence type (ST-1146), 3 isolates from water samples (Mallorca, Spain) and 1 clinical, non-CF isolate, obtained at the Son Dureta University Hospital (Mallorca, Spain), were selected to be studied by comparative genomics. These isolates can be considered a good example of close-related strains to study microevolution. ST-1146 has the allelic profile 5-11-57-33-1-6-3 for the seven genes *acsA*, *aroE*, *guaA*, *mutL*, *nuoD*, *ppsA* and *trpE*, which were established by Curran et al. [22] for a *P. aeruginosa* MLST study and is available in the *P. aeruginosa* MLST database (http://www.pseudomonas.com/). MLST is the reference method for typing clinical strains of *P. aeruginosa* and other bacteria. The environmental isolates could be differentiated from the clinical isolate by the antibiotic susceptibility profile according to the Magiorakos et al. [23] classification. The environmental isolates were non-multidrug resistant (non-MDR), and the clinical isolate was multidrug resistant (MDR), resistant to aztreonam, ceftazidime, imipenem and piperacillin-tazobactam. The main purpose of this study is to fill the gap between precise genomic studies of clinical strains and widespread studies on environmental strains by studying in detail genomes from isolates of the same ST from both origins, clinical and environmental. The genomic analysis of these four isolates was focused on four main aspects: a) the presence of exclusive and differentiating genes; b) the presence of nucleotide substitutions when compared with strains *P. aeruginosa* PAO1-UW and UCBPP-PA14 as references (the allele distribution in gene categories, allelic profile comparisons) and the presence of nucleotide substitutions among ST-1146 isolates; c) the analysis of genes that are involved in pathogenicity (virulence factors, specific killing regions, lung infection potential, or pyocin genes); and d) contribution of phages and CRISPRs (Clustered Regularly Interspaced Short Palindromic Repeats) sequences in isolates differentiation.

## Materials and Methods

### *P. aeruginosa* isolates

Environmental *P. aeruginosa* isolates were isolated on Cetrimide Agar (Merck) as the selective medium from 2 subsurface water samples taken from the same well in Santa Margalida (Mallorca, Spain). One sample was taken in October 2010 (isolate P37), and the second sample was taken in February 2011 (isolates P47 and P49). The clinical isolate (SD9) was isolated on MacConkey agar plates (bioMérieux) at 37°C from a patient's ulcer. The four isolates were assigned to ST-1146 following previously described methods [24].

### *De novo* assembled genome analysis

The draft genome sequence of the P37, P47, P49 and SD9 isolates were obtained using the reads from Illumina HiSeq 2000 paired-end libraries. The reads were *de novo* assembled using the Newbler Assembler v2.7 program (Roche). Drafts were annotated using the NCBI Prokaryotic Genome Annotation Pipeline (PGAP). The Whole Genome Shotgun projects have been deposited at DDBJ/EMBL/GenBank under the accessions AMVN00000000 (SD9), AMVO00000000 (P37), AMVP00000000 (P39) and AMVQ00000000 (P49). The gene and protein prediction for the ST-1146 isolates were performed using the Metagenemark program [25]. Protein clustering analysis was initially performed with strains P37, P47, P49 and SD9 and later including two *P. aeruginosa* reference genomes, PAO1-UW (NC_002516.2)(PAO1) and UCBPP-PA14 (NC_008463.1) (PA14), using the Cd-hit program available at Cd-hit home page [26]. The protein predictions from the two reference genomes were obtained from the NCBI database. Two proteins with at least 50% sequence identity over at least 50% of the protein length were considered to belong to the same gene family. Proteins exclusive of each strain and those shared between two or more strains were counted and represented in Venn diagrams. UPGMA dendrogram were used to compare the amino acid sequences distances. The isolate-specific genes were deeply analyzed. Their functions were established comparing with the sequences from the NCBI database. BAGEL2 program was used for specific detection of pyocines [27].

Furthermore, the isolate-specific proteins of this study were compared by local BLAST with the exclusive predicted proteins obtained by Grosso-Becerra et al. [21] in their study of 17 *P. aeruginosa* strains isolated from different habitats (water, plant, human origins).

### Genome comparisons

A BLAST Matrix was calculated to display the conserved gene families in a set of 25 *P. aeruginosa* genomes and the 4 isolates (P37, P47, P49 and SD9) from our study, using the Biotools for Comparative Microbial Genomics (CMG-Biotools) [28]. All complete *P. aeruginosa* genomes available in the NCBI database were used: YL84 (CP007147.1), PAO1 (NC_002516.2), UCBPP-PA14 (NC_008463.1), PA7 (NC_009656.1), LESB58 (NC_011770.1), M18 (NC_017548.1), NCGM2.S1 (NC_017549.1), DK2 (NC_018080.1), B136-33 (NC_020912.1), RP73 (NC_021577.1), PA1R (NC_022808.1), PA1 (NC_022806.1), MTB-1 (NC_023019.1), LES431 (NC_023066.1), SCV20265 (NC_023149.1), PACS2 (NZ_AAQW01000001.1), 39016 (NZ_AEEX01000000), 19BR (NZ_AFXJ01000001.1), 213BR (NZ_AFXK01000001.1). Additionally, the *P. aeruginosa* draft genomes of strains 148 (ATAJ00000000), 2192 (AAKW00000000), C3719 (AAKV00000000), ID4365 (ATAI00000000), IGB83 (ATAH00000000) and M10 (ATAG00000000) were downloaded in its last version from NCBI database and included in the analysis. Two proteins with at least 50% sequence identity over at least 50% of their length were considered as belonging in the same gene family. The similarity percentage between the genomes were then sorted into a distance matrix and then plotted as a UPGMA dendrogram, using the Pearson coefficient implemented in the PermutMatrix program [29]. The sequence types were established for all genomes according to Currant et al. [22], using the *Pseudomonas aeruginosa* MLST Database (http://pubmlst.org/paeruginosa/).

### Genome mutational profiles

The original Illumina reads of the 4 genomes sequenced were processed using the GS Reference Mapper software package version 2.6 (Roche Inc). High quality Illumina sequencing reads, sequences with more than 100× reads coverage, were aligned using both *P. aeruginosa* PAO1 and PA14 as reference genomes. Variants with respect to both reference sequences were identified with the GS Reference Mapper v2.7 (Roche) software (AllDiff and HCDiff reports). The presence of variant candidates (alleles) were detected using the high-confidence method (i.e., observed in >80% of the reads). The detected variants and positions were extracted for the purpose of comparison from the HCDiff output file. The GFF files obtained from the *Pseudomonas* Database [30] from both reference genomes were used to annotate and count variants to generate the mutational gene profiles. The exclusive alleles detected for each strain were classified in 27 functional categories according to the PseudoCap functional classification [30].

### Polymorphic sites and nucleotide substitutions

The contigs obtained with the original Illumina reads mapped against the *P. aeruginosa* PAO1 genome were aligned with the Mauve program [31]. For that purpose, the sequences located at the ends of the contigs and the sequences not present in all five genomes were discarded. Polymorphic sites between P37, P47, P49 and SD9 were calculated from this alignment with the DnaSP package, version 5.0 [32] and were localised along the chromosome.

### CRISPR analysis

The CRISPRFinder at the CRISPRs web server (http://crispr.u-psud.fr) was used to identify the Clustered Regularly Interspaced Short Palindromic Repeats (CRISPRs) elements [33] in the *de novo* assembled genomes. The CRISPRdb database [34] and CRISPRcompar [35] tool, both available at the CRISPRs website were used to display and compare the CRISPRs. A *Pseudomonas* phages database was constructed by downloading from the NCBI the corresponding FASTA files (ftp://ftp.ncbi.nih.gov/genomes/Viruses). The final database was formatted and interactively searched with BLAST in the context of the UGENE [36] software.

## Results

### Whole genome characteristics of strains in ST-1146

The Illumina reads obtained for the four strains in ST-1146 were *de novo* assembled and the draft genome annotated. The main features for each assembly and annotation prediction are provided in Table S1. The number of contigs ranged from 112 to 161. In all of the isolates, the percentages of bases with a consensus quality score of at least 40 (Q40), were higher than 99.9%. Based on the MetaGeneMark annotation prediction, between 5841 and 5873 ORFs were detected in the environmental isolates, and 5972 ORFs were detected in the clinical isolate SD9.

### Genome comparisons

The 29 strains analyzed shared 4106 gene families in their core genome from a total of 10473 gene families in the pangenome. The analysis of the 29 genomes studied is represented in a dendrogram (Figure S1). Minimal percentage of shared proteins was detected with strain PA7 (range 68.9–78.4%) and maximal values were found among groups of isolates classified in the same ST: ST-146 (98.9%), ST-235 (86.0%), ST-277(98.1%), ST-782 (97.0%) and ST-1146 (95.0–97.9%). This last ST was the only one with environmental and clinical isolates and the four isolates clustered in a well differentiated branch in the dendrogram.

### Gene comparisons with Cd-hit

**Figure 1:**
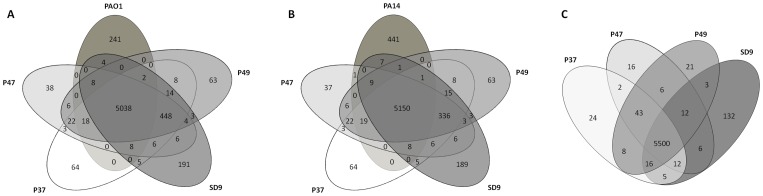
Venn diagram showing the number of shared and exclusive genes of isolates. Number of genes of isolates P37, P47, P49 and SD9 based on the Cd-hit results are shown, referred to A) *P. aeruginosa* PAO1; B) *P. aeruginosa* PA14; C) ST-1146 isolates.

Cluster comparisons of the four isolates in ST-1146 alone and with the reference genomes PAO1 and PA14 were performed using the program Cd-hit. Venn diagrams were generated to visualise the cluster distributions (Fig. 1). A total of 6200 or 6403 clusters were determined when PAO1 or PA14 were included in the analysis. As depicted in Fig. 1, 5038 genes were shared by the 4 strains of ST-1146 and PAO1, and 5150 genes were shared with PA14. Four-hundred and forty-eight protein clusters were present in the isolates of ST-1146 and not in PAO1, and 241 proteins were PAO1 strain specific (Fig. 1A). Strain-specific proteins in the ST-1146 strains when PAO1 was included in the analysis were 241, 191, 64, 38 and 63 in strains PAO1, SD9, P37, P47 and P49, respectively. When strain PA14 was compared with the strains of ST-1146, strain-specific proteins were 441, 189, 64, 37 and 63 in strains PA14, SD9, P37, P47 and P49, respectively (Fig. 1B).

The four sequenced genomes of the ST-1146 isolates shared 5500 protein clusters from the total of 5959 analysed (Fig. 1C). The number of proteins shared by the three environmental strains was slightly higher (5543 proteins). The isolate-specific genes found in internal regions of the contigs were 132, 24, 16 and 21 genes in isolates SD9, P37, P47 and P49, respectively. Some isolate-specific genes were located at the beginning or at the end of a contig (61 in SD9, 37 in P37, 20 in P47 and 39 in P49), which represent 0.4–1% of the total number of genes. These genes were incompletely sequenced and they were not included in the analysis. The isolate-specific proteins exhaustively analysed for the four strains are shown in Table S2. Three genes of isolate P37, related to insertion sequences from the IS1, IS3 and IS5 families, were not found in the other genomes. One gene of SD9 was related to bacteriophage Pf1 (hypothetical protein, gene_id 2506), and other 55 genes related to bacteriophages F116 and/or H66 were not found in the other genomes and will be discussed in a specific section.

### Genome mutational profiles and allele comparisons

#### 1. Nucleotide substitutions in the coding genes

According to mutational profiling (Tables S3 and S4), the nucleotide substitutions in the coding genes of strains in the ST-1466 genomes compared with PAO1 were 49931 in SD9, which was higher than in the environmental strains P37, P47 and P49 (49545, 49444 and 49578, respectively). Compared with PA14, the number of substitutions was lower than with PAO1: 32387 in SD9, 31982 in P37, 31964 in P47, and 32045 in P49. From a total of 5565 genes present in strain PAO1, 5126 genes with nucleotide substitutions were detected in ST-1146 isolates. ST-1146 isolates shared 4506 (63.7%) identical genes: 4067 genes identical among them but different to PAO1 and 439 genes identical between them and PAO1. From a total of 5892 genes in PA14, 4841 genes presented nucleotide substitutions in ST-1146 strains, 1051 genes were identical among the four strains and PA14.

#### 2. Isolate-exclusive alleles

**Figure 2:**
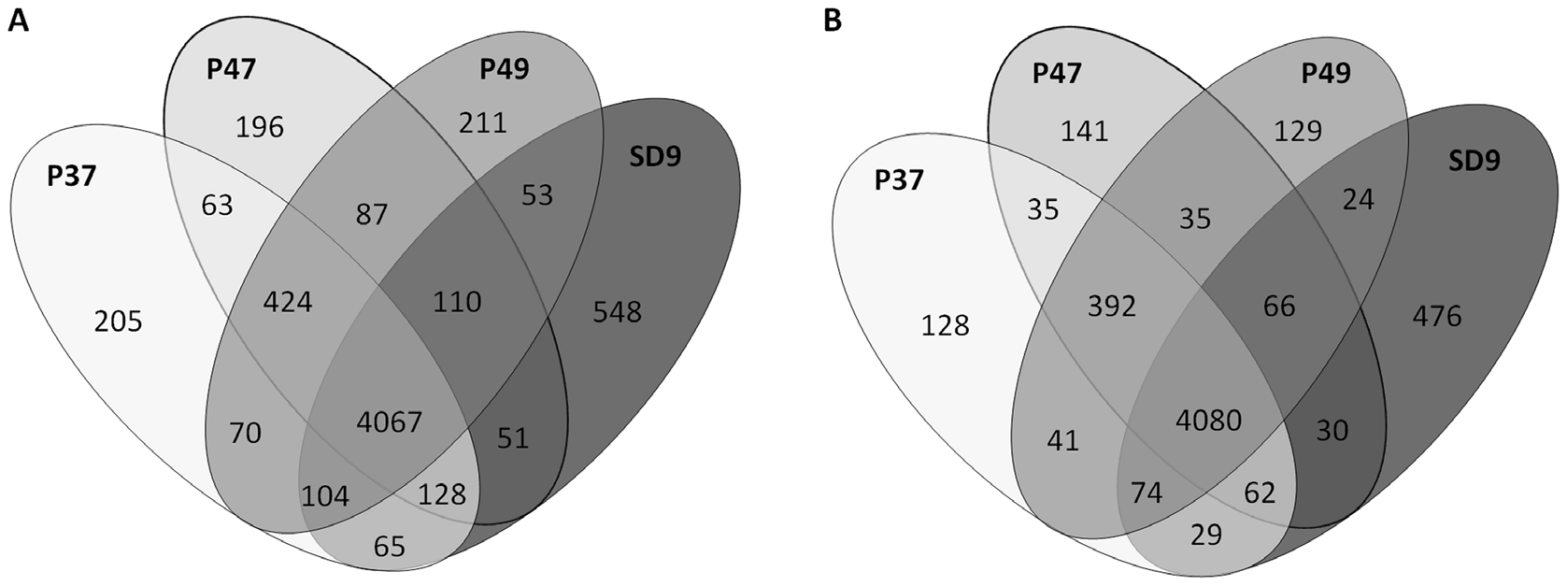
Venn diagram showing the number of shared and exclusive alleles of isolates. Number of alleles of P37, P47, P49 and SD9 referred to A) *P. aeruginosa* PAO1; B) *P. aeruginosa* PA14.

The alleles exclusive to each isolate and the alleles shared between two or more isolates obtained from the two mutational profiles of ST-1146 were counted and represented in Venn diagrams (Fig. 2). The allele number compared to PAO1 and present in only one strain and not in others was as follows: isolate P37, 205 alleles (4.0%); isolate P47, 196 alleles (3.8%); isolate P49, 211 alleles (4.1%); and finally, isolate SD9, 548 alleles (10.7%). The number of alleles exclusive to strain SD9 (548) was 2.5 times higher than the exclusive alleles in the environmental strains, which shared 424 alleles not present in SD9. Compared to PA14, the allele numbers present in only one isolate were slightly lower, although isolate SD9 presented the highest number (476) of isolate-exclusive alleles (an average of 3.6 times higher than the environmental strains).

#### 3. Functional categories

**Figure 3:**
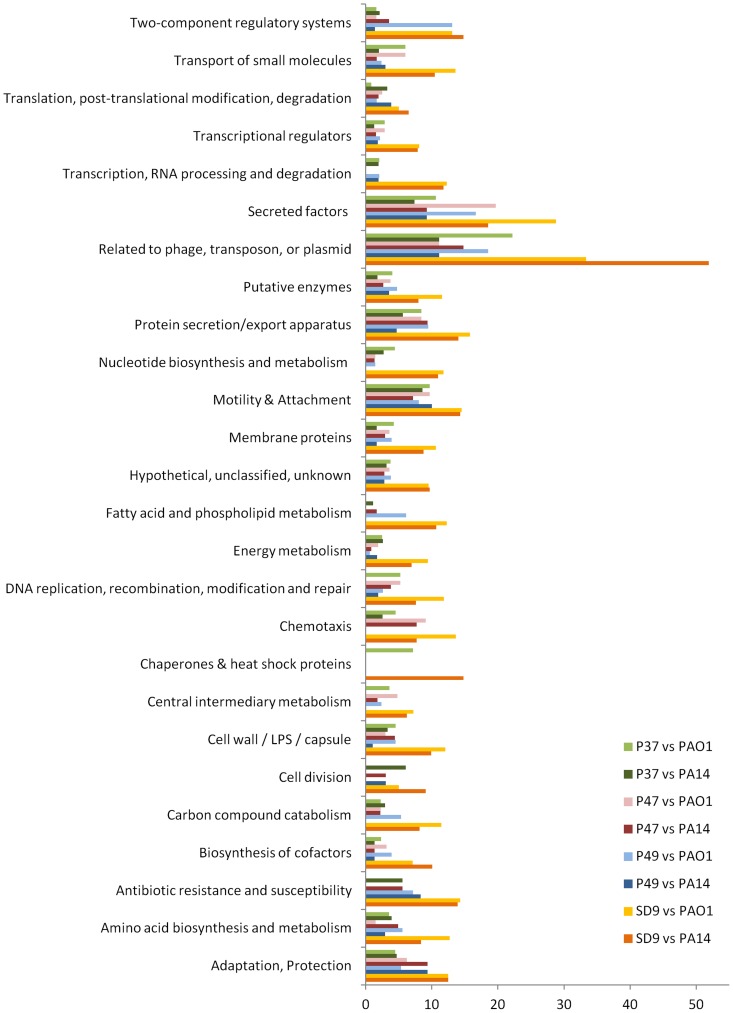
PseudoCAP functional classification of the exclusive alleles of isolates P37, P47, P49 and SD9. Comparison of the exclusive alleles of isolates P37, P47, P49 and SD9 with *P. aeruginosa* PAO1 and *P. aeruginosa* PA14.

All the genes of the isolates in ST-1146 studied in the mutational profile were grouped by functional categories (Fig. 3, Tables S5 and S6). All of these data are related to the total number of genes that are not identical to PAO1 and PA14 in each category, and the ratio among the alleles was calculated (Fig. 3, Tables S5 and S6). Not considering the categories “Hypothetical, unclassified, unknown” and “Putative enzymes”, the genes grouped in the functional category “Membrane proteins” presented the highest absolute values of nucleotide substitutions and alleles in all ST-1146 isolates for the mutational profile compared with PAO1 and the functional category “Transport of small molecules” when compared with PA14. The percentage of exclusive alleles for each strain (p, q, r, s) in each category (n) was calculated for the genes not identical to PAO1 or PA14. In the mutational profile of PAO1 compared with ST-1146, the categories of genes “Related to phage, transposon or plasmid” and “Secreted factors” predominated in all strains (range 10–22% in the environmental isolates and 29–33% in the clinical isolate SD9). In the comparison with PA14, nucleotide substitutions in these two categories were also predominant in all cases, except for P49. For P49, the predominant categories were “Related to phage, transposon or plasmid” (11%) and “Motility and attachment” (10%).

The ratios of the different alleles between SD9 and each environmental isolate were calculated for each PseudoCap functional class (Tables S5 and S6). In isolate SD9, the ratio of exclusive alleles in most of the categories was 2–15 times higher than in the environmental strains or the comparison with PAO1 and 2–10 times higher for the comparison with PA14 (mean values of 3.3 for PAO1 and 3.9 for PA14). All 14 genes in the category “Antibiotic resistance and susceptibility” were identical in isolates P37 and P47, only one different allele was found in isolate P49, and different alleles were found in SD9 compared with PAO1. Some of these alleles were also different with PA14: 2 (P37, P47), 3 (P49) and 5 (SD9).

### Nucleotide polymorphisms

The intraclonal diversity of the members of the clonal complex ST-1146 was also studied by comparing the nucleotide sequences of the four strains and mapping the sequences against the reference genome PAO1 to determine the nucleotide polymorphic sites distribution in the isolates. The number of polymorphic sites for the 5 genomes was 56626. The total number of nucleotide substitutions was 56657; therefore, practically all of the polymorphic sites (99.95%) presented only 1 nucleotide substitution. The polymorphic sites for the four ST-1146 genomes were 1056, and the total number of substitutions was 1072. The polymorphic sites resulting from a different nucleotide in SD9, P37, P47 and P49 were 624, 147, 150 and 151, respectively. Nucleotide substitutions are shown in Table 1.

**Table 1:** Nucleotide substitutions in the polymorphic sites of the isolates P37, P47, P49 and SD9.

Nucleotide substitutions	P37	P47	P49	SD9	Total
A	33	34	34	184	285
T	32	27	25	158	242
G	40	34	43	139	256
C	39	45	44	134	262
Indel	3	10	5	9	27
Total	147	150	151	624	1072

**Figure 4:**
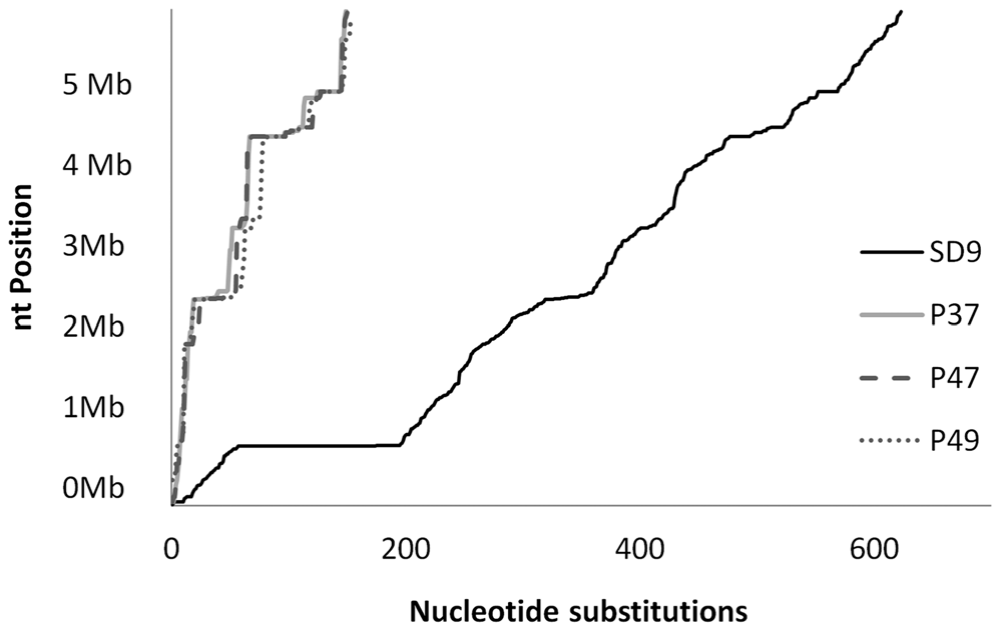
Distribution of nucleotide substitutions along the genome of the isolates P37, P47, P49 and SD9.

A representation of the nucleotide substitutions along the chromosome was created for each isolate (Fig. 4). The distribution along the chromosome was not homogenous among the four isolates. The three environmental isolates showed a similar distribution of approximately 150 substitutions. A specific region of polymorphic sites was more evident in the SD9 isolate. In SD9, 139 substitutions (22.3%) were located in an 8531 bp region of genes related to phage Pf1. When this plateau was analysed in more detail, a total of 71 substitutions were found in intergenic regions, and 68 were located in genic regions.

### Pathogenicity factors

The presence of more than 300 pathogenicity factors have been checked in the *de novo* assembled genomes of the four ST-1146 isolates.

#### 1. Virulence factors

The 265 virulence factors proposed by Wolfgang et al. [37] and the 41 virulence-related genes proposed by Feinbaum et al. [38] were present, and no significant differences were found between the virulence factors, as these genes are highly conserved: 228 genes were identical in the four ST-1146 isolates and only 19 genes presented more than one nucleotide substitution; 98% of the 306 genes coding for virulence factors presented 97–100% similarity with PAO1 (Table S3), with the exceptions of 2 genes: *wbpL* (not found) and *pilA* (low coverage with PAO1 but 99% similar with a 100% query coverage with strain PAK). The gene *exoS* was not found, and *exoU* was detected in the 4 isolates and showed a 99.4% similarity with *exoU* PA14_51530 (Table 2).

**Table 2:** Exoenzymes, pyocins and killing regions detected in the isolates P37, P47, P49 and SD9.

Genes	PAO1	PA14	ST-1146	Function
Exoenzymes				
*exoT*	PA0044	PA14_00560	PRESENT	Exoenzyme
*exoY*	PA2191	PA14_36345	PRESENT	Exoenzyme
*exoU*	ABSENT	PA14_51530	PRESENT	Exoenzyme
*exoS*	PA3841	ABSENT	NOT FOUND	Exoenzyme
Pyocins				
*pys2*	PA1150	ABSENT	PRESENT	E2 colicins
*imm2*	PA1151	ABSENT	NOT FOUND	E2 colicins
*pyoS3A*	ABSENT	PA14_ 49520	NOT FOUND	S type pyocin protein
*pyoS3I*	ABSENT	PA14_ 49510	NOT FOUND	immunity protein
*S4*	PA3866	PA14_13940	NOT FOUND	S type pyocin protein
*pyoS5*	PA0985	PA14_ 59220	NOT FOUND	Ia and Ib
*imm S5*	PA0984	PA14_59230	NOT FOUND	immunity protein
*R2*	PA0622	PA14_08070	NOT FOUND	R2 pyocin
Killing regions				
Killing regions	ABSENT	PA14_03370	NOT FOUND	Unknown
Killing regions	ABSENT	PA14_23420	NOT FOUND	O-antigen biosynthesis
Killing regions	ABSENT	PA14_23430	NOT FOUND	O-antigen biosynthesis
Killing regions	ABSENT	PA14_27680	PRESENT	Unknown
Killing regions	ABSENT	PA14_27700	PRESENT	Putative transcription regulator
Killing regions	PA4527	PA14_58760	PRESENT	*pilC*
Killing regions	ABSENT	PA14_59010	NOT FOUND	Unknown
Killing regions	ABSENT	PA14_59070	NOT FOUND	Unknown
Killing regions	PA4552	PA14_60290	PRESENT	*pilW*

The corresponding locus tag of strains *P. aeruginosa* PAO1 and PA14 are indicated.

#### 2. Specific killing regions

Lee et al. [7] defined 6 common and specific killing regions in strain *P. aeruginosa* PA14 that consist of 9 genes required for *Caenorhabditis elegans* killing. Four PA14 killing regions were present in ST-1146 isolates (Tables 2, S3 and S4) and were identical between them (Tables 2, S3 and S4). The protein PilC had 15 and 55 different amino acids when compared with the corresponding PAO1 (PA4527) and PA14 (PA14_58760) proteins (95% and 85% similarity, respectively). PilW was 99.6% similar to the corresponding protein from PAO1 (PA4552) and differed in 127 amino acids (53.5% similar from PA14 (PA14_60690) (Tables 2, S3 and S4). Two PA14 killing genes that were not present in PAO1 were also found in ST-1146 strains: one gene was identical and conserved (PA14_27680) and PA14_27700 presented 99.6% identity in all isolates.

#### 3. Lung infection genes

A list of 85 genes defined by signature tagged mutagenesis in Winstanley et al. [39] and Potvin et al. [40] was analysed in the ST-1146 isolates, and all of these genes were detected (Tables 2, S3 and S4). With the exception of only five genes (PA4226, PA2583, PA1569, PA4284 and PA5002), the remaining 80 genes were conserved among all ST-1146 isolates but were different from those present in strains PAO1 and PA14.

#### 4. Pyocins

The isolates of ST-1146 presented one type of S-pyocin: *pys2* gene with 87% nucleotide identity with PAO1 gene and was absent in PA14 (Table 2).

### Antibiotic resistance genes

Many CF isolates acquire hyper-mutator capabilities by mutations in *mutS* or *mutT* genes, increasing the mutation rate and consequently the rate of mutations coding for antibiotic resistances. Isolates of ST-1146 presented *mutS* and *mutT* genes identical between them, and 99.1% and 98.9 similar to PAO1 genes. The major efflux pumps contributing to intrinsic and mutational antibiotic resistance are coded by the operon MexAB-OprM and by the ancillary system MexCD-OprJ. Both were present, and the MexEF-OprN and the MexGHI-OpmD efflux pump was also found (Table S7). This pump confers resistance to aminoglycoside antibiotics, is required for biofilm formation, facilitates cell to cell communication and promotes virulence and growth in *P. aeruginosa*. No significant differences were found in these genes between ST-1146 isolates. The comparison of efflux pump genes with PA14 showed a considerably higher number of nucleotide substitutions in *mexD*, with 62 different nucleotides resulting in 10 different amino acids.

The *oprD* gene codes for a specialised pore protein OprD, which allows for the selective permeation of basic amino acids and their structural analogues, such as the carbapenem antibiotics imipenem and meropenem. The *oprD* gene in ST-1146 presented 134 mutations in the environmental isolates and 135 mutations in SD9, resulting in 28 different amino acids when compared to PAO1 (PA0958) and two deletions in positions 372 and 381. This gene was more similar to the one present in *P. aeruginosa* strain PA7, an atypical, phylogenetically distant, non-respiratory *P. aeruginosa* strain as depicted in the corresponding UPGMA dendrogram (Figure S2). The similarities among the ST-1146 isolates were 93%, 91% and 89% respect to PA7, PA14 and PAO1 (Table S7 and Fig. S2). The AmpC beta-lactamase gene (PA4110, PA14_10790) and the Cat chloramphenicol acetyl transferase gene (PA0706, PA14_55170) were detected with a 98–99% identity among ST1146 isolates and with PAO1 and PA14 strains.

### Phage related genes

**Figure 5:**
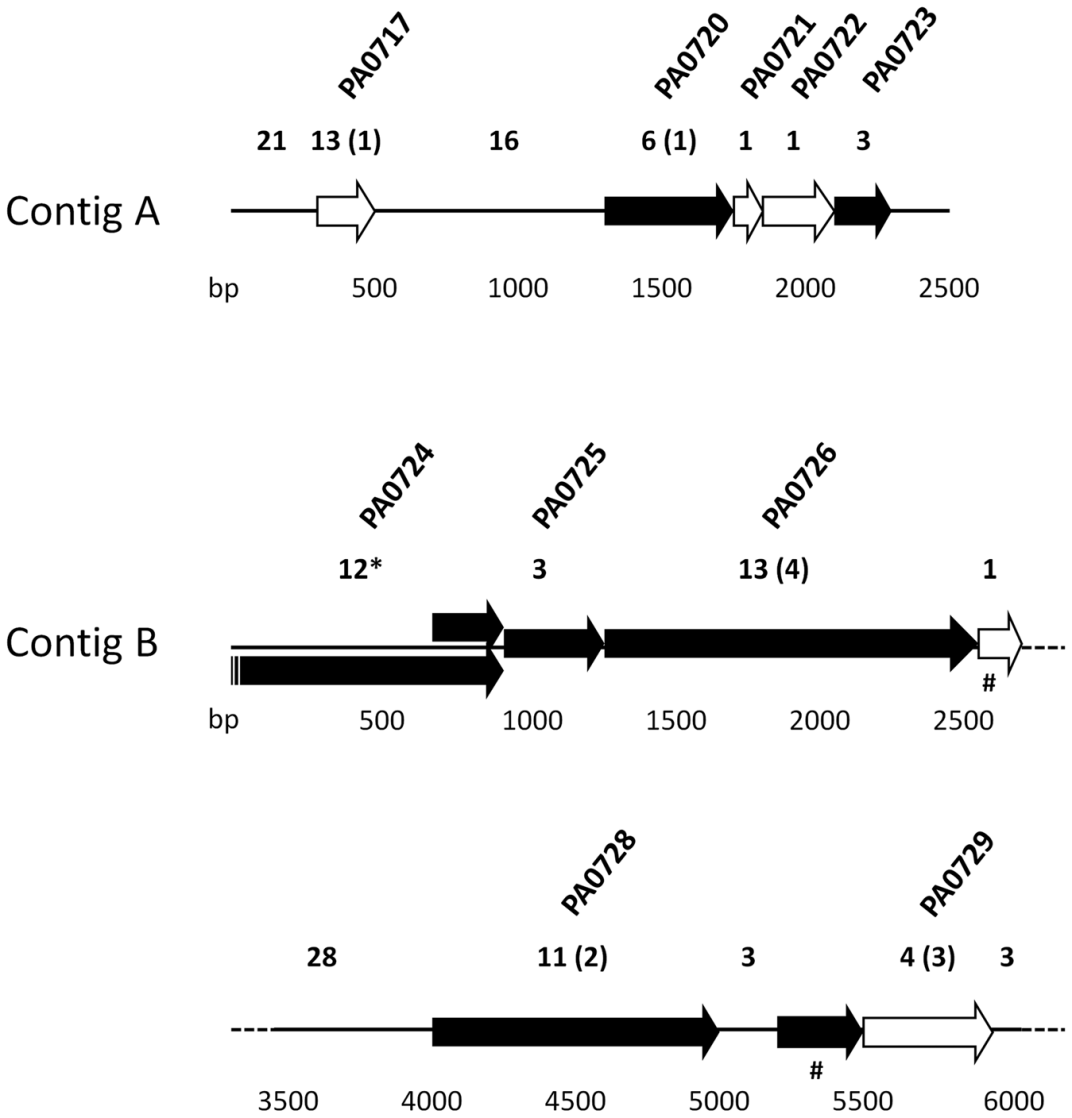
Nucleotide and amino acids substitutions in the hot spot region of original reads of SD9 mapped against PAO1. The genes related to bacteriophage Pf1 found in this region (8531 pb located in two contigs) with known function are indicated in black; the hypothetical proteins are indicated in white. The number of polymorphic sites located in intragenic and genic regions are indicated below each region. The number of amino acids substitutions in the protein of the 3 environmental isolates are indicated in brackets. *shorter protein in SD9 as a result of a deletion which produces a stop codon. bp:base pair. # ORFs detected in ST-1146 and not in PAO1, locus tag in SD9: C531_00040 and C531_14921.

The accessory genome is central to *P. aeruginosa* biology as a primary contributor to the genome evolution. The presence of phages and phage-like elements are considered to be reservoirs of genetic diversity. A high number of polymorphic sites were detected between SD9 and the environmental strains in the Pf1 gene region (Fig. 5, Table 3). In the *de novo* assembled genomes these genes were found in several contigs (5–6 contigs) in all ST-1146 isolates. All genes, with the exception of the first hypothetical protein (PA0717), could be located in the same order as in PAO1. PA0717 hypothetical protein was located in all ST-1146 isolates in a contig flanked by other genes not related to phage Pf1. Twelve phage proteins related to Pf1-like phages (Pf4) were detected in ST-1146 isolates together with the integrase (PA0728) as is described in *P. aeruginosa* LESB58 [39] or other clinical *P. aeruginosa* strains [41]. A local BLAST from Pf1 genes of SD9 against PA14 genome showed that Pf1 genes are present, with the exception of the hypothetical protein PA0729 that was not present and the integrase gene (PA0728) showed a low similarity value (52%).

**Table 3:** Nucleotide and amino acids substitutions in the genes related to phage Pf1 in isolate SD9 compared with the environmental isolates.

Gene	Locus tag in PAO1 genome	Length (bp)	No. of nucleotide substitutions	No. of amino acids substitutions	Aminoacid substitutions		
					Position	SD9	P37, P47, P49
Hypothetical protein of bacteriophage Pf1	PA0717	213	13	1	26	Ile	Gly
Helix destabilizing protein of bacteriophage Pf1	PA0720	435	6	1	121	Ala	Thr
Hypothetical protein of bacteriophage Pf1	PA0721	93	1	0			
Hypothetical protein of bacteriophage Pf1	PA0722	252	1	0			
Coat protein B of bacteriophage Pf1	PA0723	240	3	0			
Coat protein A of bacteriophage Pf1	PA0724	207	12	0			
Hypothetical protein of bacteriophage Pf1	PA0725	357	3	0			
Zona occludens toxin	PA0726	1275	13	4	254	Ala	Gly
					335	Glu	Asp
					341	Arg	His
					382	Ala	Pro
Hypothetical protein of bacteriophage Pf1	Not found	102	1	0			
Bacteriophage integrase	PA0728	984	11	2	63	Leu	Val
					238	Leu	Ile
Prevent-host-death family protein	Not found	252	0				
Hypothetical protein	PA0729	348	4	3	33	Phe	Leu
					73	Thr	Ser
					89	Ala	Val

**(bp)** base pairs.

**Figure 6:**
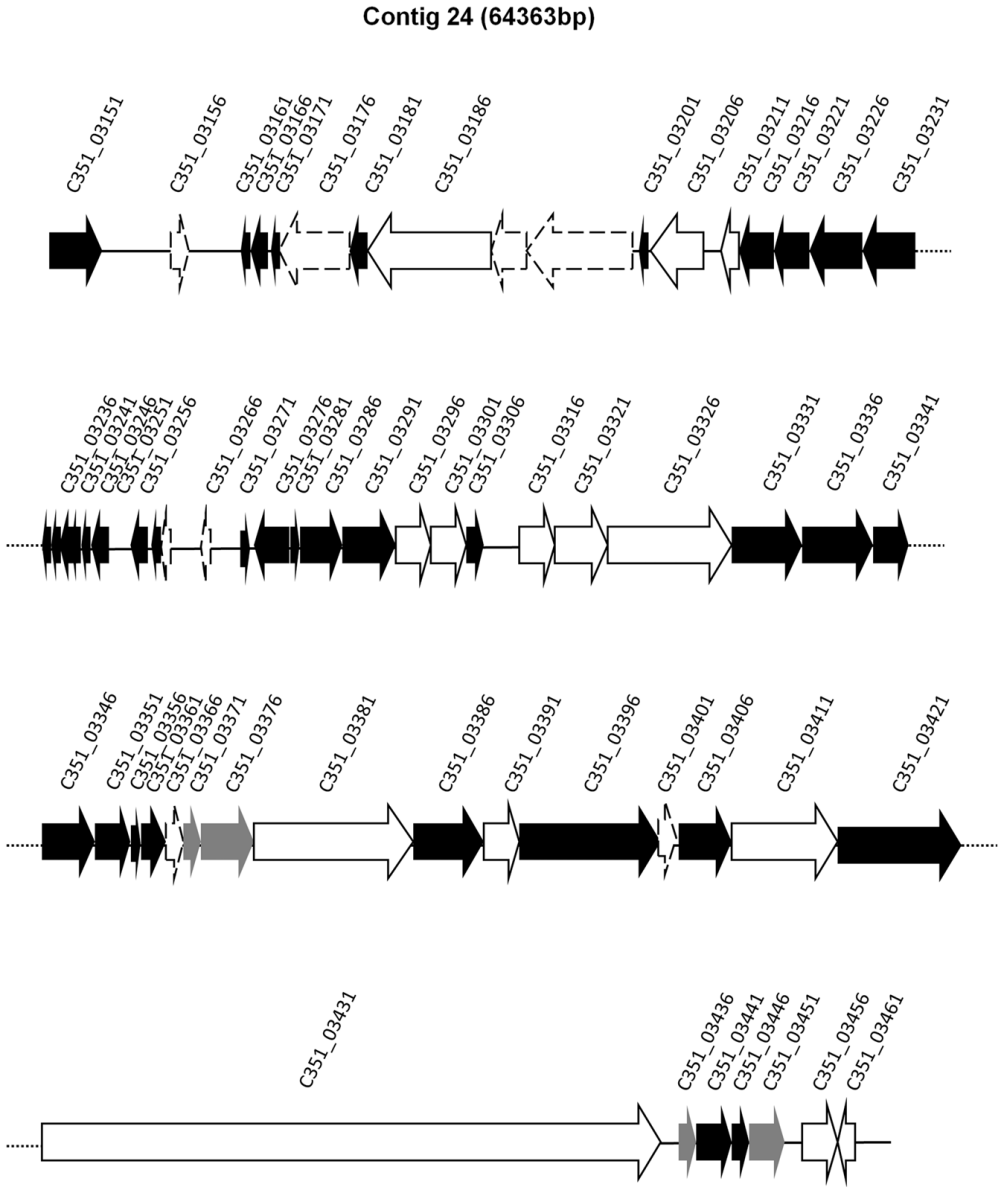
Gene map of phage present in SD9 and not present in the environmental isolates. Genes which first hit in BLAST was *P. aeruginosa* phage F116 are indicated in black, genes which first hit was *Pseudomonas* phage H66 are indicated in white. Those genes which its first hit was both phages with the same similarity and coverage percentage are indicated in grey. Genes not found in NCBI database are indicated with a discontinuous line.

When the exclusive cluster of proteins based on the Cd-hit analysis of each of the isolates was studied, the clinical strain SD9 presented exclusive CDS in a contig of 64363 bp related to previously described *P. aeruginosa* phages: F116 (65195 bp, 70 genes) and H66 (65270 bp, 71 genes). A local BLAST analysis of this contig indicated that phage F116 was 98% similar, with 76% coverage, and phage H66 was 98% similar with 78% coverage. In total, 55 of 70 genes presumptively belonging to phage similar to F116 or H66 phages were detected. A schematic representation of the phage is presented in Fig. 6, and the relationship of the genes is shown in Table S2. The exclusive phage genes of ST-1146 were compared with the exclusive protein database of the 17 *P. aeruginosa* genomes studied by Grosso-Becerra et al. [21]. Forty-two proteins similar to phage F116 were detected in the database: 35 of them were considered exclusively associated to a water strain (*P. aeruginosa* ID4365) and 7 proteins were from unknown origin (Table S8).

### CRISPRs

**Figure 7:**
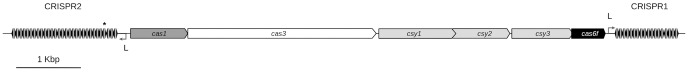
The CRISPR-Cas system detected in the ST-1146 isolates. The system was constituted by the following elements: CRISPR2, contains 29 spacers in the environmental isolates and 30 in SD9 (additional spacer indicated by*); Cas1 (*cas1*) endonuclease; nuclease/helicase Cas3 (*cas3*); 3 associated proteins Csy1 (*csy1*), Csy2 (*csy2*) and Csy3 (*csy3*) and a CRISPR-associated endonuclease Cas6/Csy4 encoded by the *cas6f* gene. The spacers (32 bp) are represented by gray rectangles between black triangles (repeats of 28 bp). The additional spacer found in the CRISPR2 of SD9 is labelled by an asterisk. Two potential leader sequences (L) of up to 244 bp are represented in front of both CRISPR loci.

Three CRISPR repetitive sequences (CRISPR1, CRISPR2 and CRISPR3) were detected in the 4 *Pseudomonas* isolates of ST-1146. The 3 CRISPRs showed a 28 bp conserved repeat consensus sequence (5′-GTTCACTGCCGTATAGGCAGCTAAGAAA-3′). The differences were mainly detected in the number of spacers, which represent potentially captured DNA. As explained in Figure 7, the CRISPR1 and CRISPR2 were similar and conserved in sequence and organization throughout the 4 isolates. Both regions were arranged in close proximity in the same contig and flanking a series of a conserved CRISPR-associated (*cas*) genes system. This CRISPR-Cas system could be included in the type I, subtype I-F (Ypest or CASS3) according to the basis of composition and structure detected [42]. The spacers of CRISPR1 and 2 had an average length of 32 bp. CRISPR1, with 18 spacers, was identical in ST-1146 isolates, but CRISPR2 showed 29 spacers in the environmental isolates and an additional spacer in isolate SD9 occupying the fourth position after a region lacking an open reading frame that follows the first locus associated to *cas* genes (*cas1*). The additional spacer of SD9 showed full identity with short stretches (11–14 nucleotides length) of several *Pseudomonas* phages (LUZ27, YuA, M6). Finally, CRISPR3 was found in a different contig, was not *cas*-associated, and with 18 spacers in the environmental isolates, was conserved in terms of sequence and arrangement. Again, the genome of isolate SD9 contained an additional spacer that showed homology with short stretches of 11 bp with different *Pseudomonas* phages (LUZ24, LUZ19, D3112, 201phi2-1, EL, D3, among other phages).

Strain PAO1 has no CRISPR systems, but some features in common were found among those of strain PA14 and ST-1146 isolates. The 2 CRISPRs of PA14 corresponded also to a type I, subtype I-F (Ypest or CASS3) structure and shared the same 28 bp repeat consensus sequence detected in the 4 isolates of ST-1146. One of the CRISPR of PA14 had 21 spacers with no homologous in the isolates of ST-1146, and no *cas* gene system was detected in its proximity. Conversely, the second CRISPR of PA14 was found in the vicinity of a complete CRISPR-Cas system, closely linked to the *cas6f* gene. This second CRISPR had up to 14 spacers from which just one was present in the CRISPR1 of the 4 isolates of ST-1146.

The detailed comparison of the different genes forming the *cas* system between PA14 and its homologous in ST-1146 isolates showed several levels of conservation. The *cas1*, *csy3* and *cas*3 genes presented 6 (from 975 bp), 10 (from 1029 bp) and 18 (from 3231 bp) nucleotide substitutions respectively, resulting in 3, 2 and 9 amino acid changes in the protein sequence. The *csy1* gene accumulated 58 nucleotide changes (from 1305 bp) in *P. aeruginosa* PA14 (20 amino acid changes) when compared with the same sequence in the ST-1146 isolates. The *csy2* and *cas6f* genes of ST-1146 were more conserved. The *csy2* gene showed 4 nucleotide changes in *P. aeruginosa* PA14 (from 984 bp), without affecting the translated Csy2, and no nucleotide change was found in *cas6f* gene (564 bp).

## Discussion

In this paper, for the first time 4 *P. aeruginosa* genomes of the same sequence type (S-T1146) have been studied by comparative genomics. Two of the water isolates were recovered from the same water sample and another was isolated 4 months earlier from the same habitat. The fourth was a clinical isolate. The main objective of our study was to assess the genomic differences and similarities between closely related strains of the same sequence type. The study of the three water isolates will allow the assessment of the microdiversity within the same population and the clinical isolate SD9 can provide insights into the adaptation process to a total different habitat. The interest of studying subpopulations of the same bacterial species has been highlighted recently by Kashtan et al. [43].

Whole genome comparisons based on CMG Biotools and Cd-hit methods demonstrated that members of ST-1146 were genomically closely related. Moreover, the environmental isolates were more closely related among themselves than to the clinical isolate, probably due to the adaptation to different habitats. These isolates can be differentiated not only by the gene content but also by the alleles of the shared genes. With the Cd-hit method and the mutational profile analysis, strain PA14 is more closely related to the ST-1146 isolates than PAO1. This is in accord with previous analyses based on multilocus sequence typing and on ANIb analysis (data not shown). Notably, PA14 is located in a separately SNPs group of PAO1 [20] and considered significantly more virulent than PAO1 [44], [45].

Two phenotypic typing methods have been classically applied to discriminate *P. aeruginosa* strains: antibiotic profiles and pyocin typing. Isolate SD9 is multidrug resistant (MDR), and OprD is responsible for the selective permeation to carbapenem antibiotics. Pirnay et al. [3] found 21 different defective *oprD* mutations conferring resistance to carbapenem antibiotics in clinical strains (CF and non-CF), and none these mutations were present in the strains of that study. Members of narrow clonal complexes often show identical *opr*D sequences [3]. In our study, the 3 environmental isolates showed identical nucleotide sequences for *oprD*, and SD9 differed by only 1 nucleotide. Interestingly, the environmental isolates were sensitive to imipenem while SD9 was resistant (data not shown). However, alterations in OprD are not the only mechanism for resistance against carbapenem antibiotics [46]. Different types of bacteriocins have been described in *P. aeruginosa*, and bacteriocin typing (production or sensitivity) has been proposed for intraspecies differentiation. All ST-1146 isolates presented the same conserved *pys2* gene.

The differences among strains should be considered with respect to not only what genes or alleles are present but also how efficient these genes are regulated or expressed. A high number of transcriptional regulators (at least 437) and two-component regulatory systems (62) were found in the isolates of ST-1146 (9% of the total genes), which reveals the complexity of *P. aeruginosa* metabolic regulation. Similarly in a previous work, 9.4% of *P. aeruginosa* genes were also considered to be involved in regulation, but only 5.8% of the genes in *Escherichia coli* [47]. The differences in the allelic profile in these 2 PseudoCap functional categories between the 4 isolates might indicate adaptation to the habitat. The environmental strains share more alleles in common in both categories than with SD9.

Single nucleotide substitutions have been studied in closely related strains isolated from cystic fibrosis patients in Germany (RN3) and in California (PA14) [10]. In RN3, 231 single nucleotide substitutions (SNPs) were reported to PA14. The authors suggested that the genes present in RN3 could provide a selective advantage to adapt and persist in CF, accumulating SNPs similar to those present in *retS* (major transcript regulators), *mexH* (encoding efflux pumps), *pvdD* (siderophore), *cndS* (cyanide) or *phnA* (quinolone). These 5 genes are present in all isolates of ST-1146, although only *retS* and *pvdD* presented nucleotide substitutions. The amino acid change (alanine, A, by threonine, T) reported in RetS in clon C is considered an adaptive mutation during a chronic infection in CF airways [8] and was also found in isolate SD9 (position 190) of ST-1146. The environmental isolates of ST-1146 and strains PAO1 and PA14 maintain an A in the same position. The other amino acid substitution in clon C (arginine, R by tryptophan, W) [8] was not found in any of the ST-1146 isolates. The MexH protein was identical in ST-1146 isolates, but different from the corresponding PA14 protein. In our study, MexD was identical in all of the ST-1146 isolates, accumulating a high number of amino acid substitutions (10) compared with PA14 (PA14_60830). It seems that the intraclonal diversity did not evolve by random drift, but was driven by selective forces that do not affect the same genes in different strains of *P. aeruginosa*.

The category of genes “Related to phage, transposon or plasmid” presented a relevant number of nucleotide substitutions. All genes coding for phage Pf1 proteins were found in the ST-1146 strains, and the corresponding alleles were identical in the environmental strains but were different from those present in SD9. The prophage Pf1 genes were found in other *P. aeruginosa* strains, as PA14 and RN3 and the Pf1-like genes are considered to be the major mutation hot spot and the most rapidly evolving part of the genome with 87 SNPs (PA14_48890-PA14_49000) [10]. This hot spot was also present in our isolates. Isolate SD9 showed a higher number of nucleotide substitutions than the environmental isolates. These data imply that the specific affected loci were subjected to the same diversifying selection pressure in the environmental isolates but not in the corresponding Pf1 genes of the clinical isolate.

The main difference in gene content between the environmental isolates and SD9 are the genes similar to the phage F116/H66 genes, which were present only in the clinical SD9. These genes are not present in the PA14 or PAO1 strains but 35 proteins are present in *P. aeruginosa* ID4365 isolated from ocean water. The presence of genes related to the phage F116/H66 in SD9 reflects the genome plasticity of the studied isolates, which have adapted to different environments. The presence of a Pf1 hot spot and the fact that the prophage islands are critical determinants of *in vivo* competitiveness [39] could justify the high number of nucleotide substitutions detected in the clinical isolate SD9 but not in the environmental isolates. A high percentage of different alleles in the gene category “Related to phage transposon or plasmids” were also found in the environmental isolates. Horizontal gene transfer (HGT) may play a more important role than point mutations in the adaptation of *P. aeruginosa* [48]. The detection and characterization of the spacers contained among CRISPR elements could be of significance in providing insights into the evolutionary history of the bacterial isolates of ST-1146. The spacers could store, in-between the conserved repeated elements, the record of the succession of different episodes in which the microorganism had to defend against the infection of foreign genetic material (basically phages). The 4 isolates of ST-1146 could have been originated from the same clone since they have almost the same CRISPR composition. The isolate SD9 presents two additional and unique spacers in 2 of the 3 CRISPRs detected in the 4 ST-1146 isolates, which can be the result of the infection of foreign genetic material in its adaptation from the aquatic to a new habitat. Alternatively, the similarities between the CRISPRs in the environmental isolates could be the result of HGT among strains occupying the same ecological niche [49], [50].

It has been argued that the ancestors of virulent bacteria and the origin of virulence determinants lie mostly in the environmental microbiota [51]. Almost all of the virulence factors, killing genes and lung infection genes detected in experimental studies [8], [37], [39] are present and highly conserved in our isolates but are different from PAO1. The high level of conservation in the 18 strains studied by Wolfgang et al. [37] agrees with our results. Our data indicate that 100% of these genes are present in the four genomes studied. The ST-1146 isolates also have *pilC* and *pilW*, which are killing genes [7] and are not present in the 4 environmental isolates studied by Wolfgang et al. [37].

*P. aeruginosa* presents a type III secretion system (T3SS), which is not located in a pathogenicity island and is considered to be an old element in the evolution of this species [47]. The exoenzymes that belong to T3SS are *exoU*, a gene associated with increased virulence that makes the strains more cytotoxic to mammalian cells [52], and *exoS*, which is considered to be the major cytotoxin required for colonisation and dissemination during infection [3]. All of the ST-1146 isolates possess *exoU* and not *exoS* confirming the previous reports that *exoS* and *exoU* are mutually exclusive.

Our isolates have the PA14 specific killing genes [7], together with the *exo*U gene. These genes are considered prototypical elements that enhance the pathogenic characteristics in the strain harbouring these genes [3] [48]. The relative conservation of the genomes of these strains implies that conserved selective pressures contributed to the evolution of these genomes in different environmental niches. The same virulence factors required for infecting humans are also required for infecting plants, worms, or insects [51]. Some authors argue that the natural eukaryotic hosts (nematodes, insects, plants and amoebas) are the relevant natural hosts in which the selection and evolution of pathogenic traits occur, and the ability to infect humans is a secondary effect of this interaction. Our results confirm the assumption of other authors that there are no specific clones selected for a specific disease (or habitat) [3], [21], [51] and that virulence is the result of a pool of pathogenicity-related genes that interact in various combinations in different genetic backgrounds [7]. The extensive conservation of virulence genes in the genomes regardless of the clinical source suggests that the disease-causing ability of this opportunistic pathogen relies on a set of highly conserved pathogenic mechanisms. The conservation of virulence gene determinants also extends to the environmental reservoir, where a population is constantly changing but conserving the necessary tools to survive [37]. The clinical isolate SD9 has no specific gene that distinguishes this isolate from the environmental isolates, although different alleles could be detected, which is most likely due to the pressure of the eukaryotic host.

The genomic comparison of the isolates of this study with strains PAO1 and PA14 led us to conclude that not all genes of the genome are subjected to the same evolutionary forces, as demonstrated by the following: (a) The genomes are highly conserved between our isolates, including those genes classified as pathogenic factors, which shows that these genes are necessary to colonise environmental and clinical habitats; although ST-1146 isolates have characteristics similar to strain PAO1, these isolates present well-known genes that enhance the pathogenic characteristics of powerful infective strain, such as PA14; (b) As demonstrated in the mutational profile, some loci are subjected to diversifying selection as an example of coevolution, as phenazines and pyoverdines, that can be adapted in the colonisation process; (c) Several genes are not commonly present in all strains, such as *exoU* or killing genes, which are present in PA14 and ST-1146 but not present in PAO1; the opposite is the case of pyocins present in PAO1 and ST-1146 and absent in PA14; (d) Some genes in ST-1146 are more similar to other *P. aeruginosa* strains (not PAO1 and PA14), *i.e.*, *oprD* gene is more similar to the corresponding of *P. aeruginosa* PA7, a *P. aeruginosa* outlier strain from the species that is not considered *P. aeruginosa* by some researchers and *pilA*, *B* and *C* are similar to *P. aeruginosa* PAK; (e) Other differences in the accessory genome are represented by the presence of phages and prophages with high mutation rates in the clinical strain, such as F166/H66 and Pf1. CRISPR elements organization and the associated genes are also an evidence of the colonization of different habitats.

As a general conclusion, we consider the strains in the species *P. aeruginosa* to constitute a monophyletic phylogenetic branch in the genus [20], [21], [53]. The sequence type isolates selected are very close-related in the whole genome comparisons. The environmental isolates are closer related among them than to SD9 in shared genes, common alleles and phage genes present. Main differences in gene content are related to phages, and in the interaction with phages detected in CRISPRs, which is a consequence of the different habitats history. No differences in virulence genes among clinical and environmental isolates are detected. The genes implicated in pathogenicity are present and conserved in the environmental strains and thus must be considered potential pathogens. The difference must rely in the regulation/expression of the genes.

## Supporting Information

Figure S1 **UPGMA dendrogram showing the distances of common genes in 29** ***P. aeruginosa*** **genomes.** Bar indicates 0.2% distance. The sequence type of each isolate is indicated. *Sequence types data not determined.
(TIF)

Figure S2 **UPGMA dendrogram of the amino acid similarities of the imipenem outer membrane porin (OprD) of several** ***P. aeruginosa*** **strains and the four ST-1146 isolates.**
(DOCX)

Table S1 **Data of sequenced genomes of ST-1146** ***P. aeruginosa*** **isolates of this study.**
(DOCX)

Table S2 **Description of the exclusive genes analysed in ST-1146 isolates.**
(XLSX)

Table S3 **Mutational profile of isolates P37, P47, P49 and SD9 compared with** ***P. aeruginosa*** **PAO1-UW.**
(XLSX)

Table S4 **Mutational profile of isolates P37, P47, P49 and SD9 compared with** ***P. aeruginosa*** **UCBPP-PA14.**
(XLSX)

Table S5 **Genes and alleles of ST-1146 compared with** ***P. aeruginosa*** **PAO1 classified by PseudoCAP Functional Categories.**
(DOCX)

Table S6 **Genes and alleles of ST-1146 compared with** ***P. aeruginosa*** **PA14 classified by PseudoCAP Functional Categories.**
(DOCX)

Table S7 **Antibiotic resistance genes analyzed between ST-1146 isolates.**
(DOCX)

Table S8 **Comparison by local BLAST of ST-1146 exclusive proteins with the exclusive protein data of** ***P. aeruginosa*** **strains from Grosso-Becerra et al., 2014.** Local BLAST of the isolates-specific proteins of ST-1146 and exclusive predicted proteins.
(XLSX)
