# Supplementary material for: Genome Analysis of Environmental and Clinical P. aeruginosa Isolates from Sequence Type-1146
Source: PLoS One. 2014 Oct 15;9(10):e107754. doi: 10.1371/journal.pone.0107754 (PMC4198096; doi:10.1371/journal.pone.0107754)
Supplement: Figure S2 — UPGMA dendrogram of the amino acid similarities of the imipenem outer membrane porin (OprD) of several P. aeruginosa strains and the four ST-1146 isolates. (DOCX) [file pone.0107754.s002.docx]

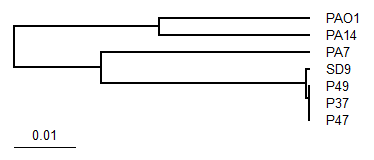


**Figure S2.** Dendrogram of the amino acid similarities of the imipenem outer membrane porin (OprD) of several *P. aeruginosa* strains and the four ST-1146 isolates by UPGMA.
