# Supplementary material for: Genome Analysis of Environmental and Clinical P. aeruginosa Isolates from Sequence Type-1146
Source: PLoS One. 2014 Oct 15;9(10):e107754. doi: 10.1371/journal.pone.0107754 (PMC4198096; doi:10.1371/journal.pone.0107754)
Supplement: Table S1 — Data of sequenced genomes of ST-1146 P. aeruginosa isolates of this study. (DOCX) [file pone.0107754.s003.docx]

**Table S1**. Data of sequenced genomes of ST-1146 *P. aeruginosa* isolates of this study.

| Data | P37 | P47 | P49 | SD9 |
| --- | --- | --- | --- | --- |
| Ecological setting | Environmental | Environmental | Environmental | Clinical |
| Source | Subsurface water | Subsurface water | Subsurface water | Ulcer |
| Origin | Well 10 | Well 10 | Well 10 | Patient 6 |
| Date | October 2010 | February 2011 | February 2011 | February 2009 |
| Resistance pattern | Non MDR | Non MDR | Non MDR | MDR |
| Number of bases assembled (bp) | 6237921 | 6236369 | 6232858 | 6301986 |
| N50 Contig Size | 108312 | 140229 | 85677 | 76889 |
| Largest Contig Size | 298247 | 499099 | 310648 | 247959 |
| Q40 Plus Bases (%) | 99.93 | 99.96 | 99.94 | 99.93 |
| Q39 Minus Bases (%) | 0.07 | 0.04 | 0.06 | 0.07 |
| GC-content (%) | 66.61 | 66.63 | 66.63 | 66.56 |
| Numbers of large contigs | 148 | 112 | 145 | 161 |
| Average contig size | 42148 | 55681 | 42985 | 39142 |
| Number of genes ^(a/b)^ | 5873/5838 | 5841/5834 | 5871/5856 | 5972/5914 |
| Number of CDSs ^b^ | 5798 | 5778 | 5806 | 5871 |
| Total CDSs size (bp) | 5579342 | 5556210 | 5577057 | 5603711 |
| Coding% | 89.4 | 89.1 | 89.5 | 88.9 |
| Average CDS length (nt)^b^ | 962 | 962 | 961 | 955 |
| tRNAs ^b^ | 37 | 53 | 47 | 40 |
| rRNA (clusters) ^b^ | 3 | 3 | 3 | 3 |
| Number of genes with assigned function ^b^ | 3887 | 3865 | 3890 | 3902 |
| Number of genes without assigned function ^b^ | 1911 | 1913 | 1916 | 1969 |

**a)** Data established with the MetaGene Mark program; **b)** Data established with the Prokaryotic Genome Automatic Annotation Pipeline (PGAAP) from the *National Center for Biotechnology Information (NCBI).*
