# Supplementary material for: Genome Analysis of Environmental and Clinical P. aeruginosa Isolates from Sequence Type-1146
Source: PLoS One. 2014 Oct 15;9(10):e107754. doi: 10.1371/journal.pone.0107754 (PMC4198096; doi:10.1371/journal.pone.0107754)
Supplement: Table S5 — Genes and alleles of ST-1146 compared with P. aeruginosa PAO1 classified by PseudoCAP Functional Categories. (DOCX) [file pone.0107754.s007.docx]

**Table S5.** Genes and alleles of ST-1146 compared with *P. aeruginosa* PAO1 classified by PseudoCAP Functional Categories.

| PseudoCAP Functional Class | Genes | | | | Alleles | | | | | | | | | | |
| --- | --- | --- | --- | --- | --- | --- | --- | --- | --- | --- | --- | --- | --- | --- | --- |
|  |  |  |  |  | P37 | | P47 | | P49 | | SD9 | | Ratio | | |
|  | m | n | o | % o/n | p | % p/n | q | % q/n | r | % r/n | s | % r/n | s/p | s/q | s/r |
| Adaptation, Protection | 3 | 112 | 27 | 24.11 | 5 | 4.46 | 7 | 6.25 | 6 | 5.36 | 14 | 12.50 | 2.80 | 2.00 | 2.33 |
| Amino acid biosynthesis and metabolism | 6 | 197 | 47 | 23.86 | 7 | 3.55 | 3 | 1.52 | 11 | 5.58 | 25 | 12.69 | 3.57 | 8.33 | 2.27 |
| Antibiotic resistance and susceptibility | 0 | 14 | 3 | 21.43 | 0 | 0.00 | 0 | 0.00 | 1 | 7.14 | 2 | 14.29 |  |  | 2.00 |
| Biosynthesis of cofactors | 10 | 127 | 23 | 18.11 | 3 | 2.36 | 4 | 3.15 | 5 | 3.94 | 9 | 7.09 | 3.00 | 2.25 | 1.80 |
| Carbon compound catabolism | 5 | 131 | 30 | 22.90 | 3 | 2.29 | 3 | 2.29 | 7 | 5.34 | 15 | 11.45 | 5.00 | 5.00 | 2.14 |
| Cell division | 3 | 20 | 1 | 5.00 | 0 | 0.00 | 0 | 0.00 | 0 | 0.00 | 1 | 5.00 |  |  |  |
| Cell wall / LPS / capsule | 7 | 133 | 26 | 19.55 | 6 | 4.51 | 4 | 3.01 | 6 | 4.51 | 16 | 12.03 | 2.67 | 4.00 | 2.67 |
| Central intermediary metabolism | 2 | 83 | 13 | 15.66 | 3 | 3.61 | 4 | 4.82 | 2 | 2.41 | 6 | 7.23 | 2.00 | 1.50 | 3.00 |
| Chaperones & heat shock proteins | 2 | 14 | 1 | 7.14 | 1 | 7.14 | 0 | 0.00 | 0 | 0.00 | 0 | 0.00 | 0.00 |  |  |
| Chemotaxis | 1 | 22 | 6 | 27.27 | 1 | 4.55 | 2 | 9.09 | 0 | 0.00 | 3 | 13.64 | 3.00 | 1.50 |  |
| DNA replication, recombination, modification and repair | 4 | 76 | 20 | 26.32 | 4 | 5.26 | 4 | 5.26 | 2 | 2.63 | 9 | 11.84 | 2.25 | 2.25 | 4.50 |
| Energy metabolism | 12 | 159 | 27 | 16.98 | 4 | 2.52 | 3 | 1.89 | 1 | 0.63 | 15 | 9.43 | 3.75 | 5.00 | 15.00 |
| Fatty acid and phospholipid metabolism | 5 | 49 | 11 | 22.45 | 0 | 0.00 | 0 | 0.00 | 3 | 6.12 | 6 | 12.24 |  |  | 2.00 |
| Hypothetical, unclassified, unknown | 209 | 1761 | 331 | 18.80 | 66 | 3.75 | 63 | 3.58 | 67 | 3.80 | 168 | 9.54 | 2.55 | 2.67 | 2.51 |
| Membrane proteins | 26 | 613 | 140 | 22.84 | 26 | 4.24 | 22 | 3.59 | 24 | 3.92 | 65 | 10.60 | 2.50 | 2.95 | 2.71 |
| Motility & Attachment | 4 | 62 | 19 | 30.65 | 6 | 9.68 | 6 | 9.68 | 5 | 8.06 | 9 | 14.52 | 1.50 | 1.50 | 1.80 |
|  |  |  |  |  |  |  |  |  |  |  |  |  |  |  |  |
|  |  |  |  |  |  |  |  |  |  |  |  |  |  |  |  |
| **Supplementary Table 2.S5** (*continuation*) |  |  |  |  |  |  |  |  |  |  |  |  |  |  |  |
| PseudoCAP Functional Class | Genes | | | | Alleles | | | | | | | | | | |
|  |  |  |  |  | P37 | | P47 | | P49 | | SD9 | | Ratio | | |
|  | m | n | o | % o/n | p | % p/n | q | % q/n | r | % r/n | s | % r/n | s/p | s/q | s/r |
| Nucleotide biosynthesis and metabolism | 4 | 68 | 11 | 16.18 | 3 | 4.41 | 1 | 1.47 | 1 | 1.47 | 8 | 11.76 | 2.67 | 8.00 | 8.00 |
| Protein secretion/export apparatus | 8 | 95 | 29 | 30.53 | 8 | 8.42 | 8 | 8.42 | 9 | 9.47 | 15 | 15.79 | 1.88 | 1.88 | 1.67 |
| Putative enzymes | 27 | 398 | 84 | 21.11 | 16 | 4.02 | 15 | 3.77 | 19 | 4.77 | 46 | 11.56 | 2.88 | 3.07 | 2.42 |
| Related to phage, transposon, or plasmid | 28 | 27 | 12 | 44.44 | 6 | 22.22 | 3 | 11.11 | 5 | 18.52 | 9 | 33.33 | 1.50 | 3.00 | 1.80 |
| Secreted factors | 5 | 66 | 31 | 46.97 | 7 | 10.61 | 13 | 19.70 | 11 | 16.67 | 19 | 28.79 | 2.71 | 1.46 | 1.73 |
| Transcription, RNA processing and degradation | 1 | 49 | 9 | 18.37 | 1 | 2.04 | 0 | 0.00 | 1 | 2.04 | 6 | 12.24 | 6.00 |  | 6.00 |
| Transcriptional regulators | 18 | 419 | 61 | 14.56 | 12 | 2.86 | 12 | 2.86 | 9 | 2.15 | 34 | 8.11 | 2.83 | 2.83 | 3.78 |
| Translation, post-translational modification, degradation | 37 | 119 | 15 | 12.61 | 1 | 0.84 | 3 | 2.52 | 2 | 1.68 | 6 | 5.04 | 6.00 | 2.00 | 3.00 |
| Transport of small molecules | 11 | 250 | 62 | 24.80 | 15 | 6.00 | 15 | 6.00 | 6 | 2.40 | 34 | 13.60 | 2.27 | 2.27 | 5.67 |
| Two-component regulatory systems | 1 | 61 | 20 | 32.79 | 1 | 1.64 | 1 | 1.64 | 8 | 13.11 | 8 | 13.11 | 8.00 | 8.00 | 1.00 |
| No classified | 0 | 1 | 0 | 0.00 |  |  |  |  |  |  |  |  |  |  |  |
| Total | 439 | 5126 | 1059 | 20.66 | 205 | 4.00 | 196 | 3.82 | 211 | 4.12 | 548 | 10.69 | 2.67 | 2.80 | 2.60 |

**m**, number of genes identical to PAO1**; n**, number of mutated genes compared with PAO1; **o**, the number of mutated genes respect to PAO1, number of genes with some different mutation between the studied strains; **p, q, r** and **s,** unique alleles.
