# Supplementary material for: Genome Analysis of Environmental and Clinical P. aeruginosa Isolates from Sequence Type-1146
Source: PLoS One. 2014 Oct 15;9(10):e107754. doi: 10.1371/journal.pone.0107754 (PMC4198096; doi:10.1371/journal.pone.0107754)
Supplement: Table S6 — Genes and alleles of ST-1146 compared with P. aeruginosa PA14 classified by PseudoCAP Functional Categories. (DOCX) [file pone.0107754.s008.docx]

**Table S6.** Genes and alleles of ST-1146 compared with *P. aeruginosa* PA14 classified by PseudoCAP Functional Categories.

| PseudoCAP Functional Class | Genes | | | | | | | Alleles | | | | | | | | | | | | | |
| --- | --- | --- | --- | --- | --- | --- | --- | --- | --- | --- | --- | --- | --- | --- | --- | --- | --- | --- | --- | --- | --- |
|  |  |  |  |  |  |  |  | P37 | | P47 | | P49 | | | SD9 | | | Ratio | | | |
|  | m | | n | | o | | % o/n | p | % p/n | q | % q/n | r | % r/n | | s | | % r/n | s/p | | s/q | s/r |
| Adaptation, Protection | 12 | | 64 | | 11 | | 17.19 | 3 | 4.69 | 6 | 9.4 | 6 | 9.38 | | 8 | | 12.50 | 2.67 | | 1.33 | 1.33 |
| Amino acid biosynthesis and metabolism | 18 | | 203 | | 34 | | 16.75 | 8 | 3.94 | 10 | 4.9 | 6 | 2.96 | | 17 | | 8.37 | 2.13 | | 1.70 | 2.83 |
| Antibiotic resistance and susceptibility | 7 | | 36 | | 11 | | 30.56 | 2 | 5.56 | 2 | 5.6 | 3 | 8.33 | | 5 | | 13.89 | 2.50 | | 2.50 | 1.67 |
| Biosynthesis of cofactors | 25 | | 149 | | 20 | | 13.42 | 2 | 1.34 | 2 | 1.3 | 2 | 1.34 | | 15 | | 10.07 | 7.50 | | 7.50 | 7.50 |
| Carbon compound catabolism | 9 | | 135 | | 17 | | 12.59 | 4 | 2.96 | 3 | 2.2 | 0 | 0.00 | | 11 | | 8.15 | 2.75 | | 3.67 |  |
| Cell division | 3 | | 33 | | 5 | | 15.15 | 2 | 6.06 | 1 | 3.0 | 1 | 3.03 | | 3 | | 9.09 | 1.50 | | 3.00 | 3.00 |
| Cell wall / LPS / capsule | 21 | | 91 | | 14 | | 15.38 | 3 | 3.30 | 4 | 4.4 | 1 | 1.10 | | 9 | | 9.89 | 3.00 | | 2.25 | 9.00 |
| Central intermediary metabolism | 7 | | 112 | | 9 | | 8.04 | 0 | 0.00 | 2 | 1.8 | 0 | 0.00 | | 7 | | 6.25 |  | | 3.50 |  |
| Chaperones & heat shock proteins | 7 | | 27 | | 4 | | 14.81 | 0 | 0.00 | 0 | 0.0 | 0 | 0.00 | | 4 | | 14.81 |  | |  |  |
| Chemotaxis | 5 | | 39 | | 6 | | 15.38 | 1 | 2.56 | 3 | 7.7 | 0 | 0.00 | | 3 | | 7.69 | 3.00 | | 1.00 |  |
| DNA replication, recombination, modification and repair | 20 | | 105 | | 16 | | 15.24 | 0 | 0.00 | 4 | 3.8 | 2 | 1.90 | | 8 | | 7.62 |  | | 2.00 | 4.00 |
| Energy metabolism | 36 | | 231 | | 26 | | 11.26 | 6 | 2.60 | 2 | 0.9 | 4 | 1.73 | | 16 | | 6.93 | 2.67 | | 8.00 | 4.00 |
| Fatty acid and phospholipid metabolism | 24 | | 178 | | 28 | | 15.73 | 2 | 1.12 | 3 | 1.7 | 0 | 0.00 | | 19 | | 10.67 | 9.50 | | 6.33 |  |
| Hypothetical, unclassified, unknown | 487 | | 1197 | | 187 | | 15.62 | 38 | 3.17 | 34 | 2.8 | 34 | 2.84 | | 116 | | 9.69 | 3.05 | | 3.41 | 3.41 |
| Membrane proteins | 16 | | 240 | | 34 | | 14.17 | 4 | 1.67 | 7 | 2.9 | 4 | 1.67 | | 21 | | 8.75 | 5.25 | | 3.00 | 5.25 |
| Motility & Attachment | 27 | | 70 | | 22 | | 31.43 | 6 | 8.57 | 5 | 7.1 | 7 | 10.00 | | 10 | | 14.29 | 1.67 | | 2.00 | 1.43 |
|  |  | |  | |  | |  |  |  |  |  |  |  | |  | |  |  | |  |  |
|  |  | |  | |  | |  |  |  |  |  |  |  | |  | |  |  | |  |  |
| **Supplementary Table S6.** (*continuation*) |  | | | | | | |  | | | | | | | | | | | | | |
| PseudoCAP Functional Class | Genes | | | | | | | Alleles | | | | | | | | | | | | | |
|  |  |  |  |  |  |  |  | P37 | | P47 | | P49 | | | | SD9 | | | Ratio | | |
|  | m | n | | o | | % o/n | | p | % p/n | q | % q/n | r | | % r/n | | s | % r/n | | s/p | s/q | s/r |
| Nucleotide biosynthesis and metabolism | 12 | 73 | | 12 | | 16.44 | | 2 | 2.74 | 1 | 1.4 | 0 | | 0.00 | | 8 | 10.96 | | 4.00 | 8.00 |  |
| Protein secretion/export apparatus | 19 | 107 | | 24 | | 22.43 | | 6 | 5.61 | 10 | 9.3 | 5 | | 4.67 | | 15 | 14.02 | | 2.50 | 1.50 | 3.00 |
| Putative enzymes | 52 | 339 | | 51 | | 15.04 | | 6 | 1.77 | 9 | 2.7 | 12 | | 3.54 | | 27 | 7.96 | | 4.50 | 3.00 | 2.25 |
| Related to phage, transposon, or plasmid | 54 | 27 | | 18 | | 66.67 | | 3 | 11.11 | 4 | 14.8 | 3 | | 11.11 | | 14 | 51.85 | | 4.67 | 3.50 | 4.67 |
| Secreted factors | 9 | 54 | | 18 | | 33.33 | | 4 | 7.41 | 5 | 9.3 | 5 | | 9.26 | | 10 | 18.52 | | 2.50 | 2.00 | 2.00 |
| Transcription, RNA processing and degradation | 7 | 51 | | 8 | | 15.69 | | 1 | 1.96 | 0 | 0.0 | 1 | | 1.96 | | 6 | 11.76 | | 6.00 |  | 6.00 |
| Transcriptional regulators | 50 | 380 | | 48 | | 12.63 | | 5 | 1.32 | 6 | 1.6 | 7 | | 1.84 | | 30 | 7.89 | | 6.00 | 5.00 | 4.29 |
| Translation, post-translational modification, degradation | 62 | 154 | | 22 | | 14.29 | | 5 | 3.25 | 3 | 1.9 | 6 | | 3.90 | | 10 | 6.49 | | 2.00 | 3.33 | 1.67 |
| Transport of small molecules | 48 | 604 | | 89 | | 14.74 | | 12 | 1.99 | 10 | 1.7 | 18 | | 2.98 | | 63 | 10.43 | | 5.25 | 6.30 | 3.50 |
| Two-component regulatory systems | 14 | 142 | | 27 | | 19.01 | | 3 | 2.11 | 5 | 3.5 | 2 | | 1.41 | | 21 | 14.79 | | 7.00 | 4.20 | 10.50 |
| No classified | 0 | 0 | | 0 | | 0.00 | |  |  |  |  |  | |  | |  |  | |  |  |  |
| Total | 1051 | 4841 | | 761 | | 15.72 | | 128 | 2.64 | 141 | 2.9 | 129 | | 2.66 | | 476 | 9.83 | | 3.72 | 3.38 | 3.69 |

**m**, number of genes identical to PA14**; n**, number of mutated genes compared with PA14; **o**, the number of mutated genes respect to PAO1, number of genes with some different mutation between the studied strains; **p, q, r** and **s,** unique alleles.
