# Supplementary material for: Genome Analysis of Environmental and Clinical P. aeruginosa Isolates from Sequence Type-1146
Source: PLoS One. 2014 Oct 15;9(10):e107754. doi: 10.1371/journal.pone.0107754 (PMC4198096; doi:10.1371/journal.pone.0107754)
Supplement: Table S7 — Antibiotic resistance genes analyzed between ST-1146 isolates. (DOCX) [file pone.0107754.s009.docx]

| ST-1146 | | | | | | Compared with PAO1 | | | | | | Compared with PA14 | | | | | |
| --- | --- | --- | --- | --- | --- | --- | --- | --- | --- | --- | --- | --- | --- | --- | --- | --- | --- |
|  | No. of different nucleotides | | | | No. of different amino  acids | No. of different nucleotides | | | | No. of different amino  acids | Locus tag | No. of different nucleotides | | | | No. of different amino  acids | Locus tag |
| Gene | P37 | P47 | P49 | SD9 |  | P37 | P47 | P49 | SD9 |  |  | P37 | P47 | P49 | SD9 |  |  |
| *mexA* | 0 | 0 | 0 | 1 | 1 | 16 | 16 | 16 | 17 | 2; 3 | PA0425 | 11 | 11 | 11 | 12 | 2; 3 | PA14_05530 |
| *mexB* | 0 | 0 | 0 | 0 |  | 17 | 17 | 17 | 17 | 1 | PA0426 | 14 | 14 | 14 | 14 | 1 | PA14_05540 |
| *mexC* | 0 | 0 | 0 | 0 |  | 6 | 6 | 6 | 6 | 4 | PA4599 | 13 | 13 | 13 | 13 | 5 | PA14_60850 |
| *mexD* | 0 | 0 | 0 | 0 |  | 12 | 12 | 12 | 12 | 2 | PA4598 | 62 | 62 | 62 | 62 | 10 | PA14_60830 |
| *mexE* | 0 | 0 | 0 | 0 |  | 6 | 6 | 6 | 6 | 1 | PA2493 | 4 | 4 | 4 | 4 | 1 | PA14_32400 |
| *mexF* | 1 | 0 | 0 | 0 | 1 | 11 | 10 | 10 | 10 | 0; 1 | PA2494 | 7 | 6 | 6 | 6 | 1; 0 | PA14_32390 |
| *mexG* | 0 | 0 | 0 | 0 |  | 2 | 2 | 2 | 2 | 1 | PA4205 | 2 | 2 | 2 | 2 | 1 | PA14_09540 |
| *mexH* | 0 | 0 | 0 | 0 |  | 9 | 9 | 9 | 9 | 2 | PA4206 | 11 | 11 | 11 | 11 | 3 | PA14_09530 |
| *mexI* | 0 | 0 | 0 | 0 |  | 3 | 3 | 3 | 3 | 1 | PA4207 | 12 | 12 | 12 | 12 | 0 | PA14_09520 |
| *mexT* | 0 | 0 | 0 | 0 |  | 12 | 12 | 12 | 12 | 33 | PA2492 | 5 | 5 | 5 | 5 | 0 | PA14_32410 |
| *mexZ* | 0 | 0 | 0 | 0 |  | 4 | 4 | 4 | 4 | 0 | PA2020 | 16 | 16 | 16 | 16 | 2 | PA14_38380 |
| *opmD* | 0 | 0 | 0 | 0 |  | 15 | 15 | 15 | 15 | 2 | PA4208 | 12 | 12 | 12 | 12 | 3 | PA14_09500 |
| *oprD* | 0 | 0 | 0 | 1 | 1 | 134 | 134 | 134 | 135 | 28; 29 | PA0958 | 124 | 124 | 124 | 125 | 29; 30 | PA14_51880 |
| *oprJ* | 0 | 0 | 0 | 0 |  | 8 | 8 | 8 | 8 | 1 | PA4597 | 12 | 12 | 12 | 12 | 3 | PA14_60820 |
| *oprM* | 0 | 0 | 0 | 0 |  | 18 | 18 | 18 | 18 | 0 | PA0427 | 1 | 1 | 1 | 1 | 0 | PA14_05550 |
| *oprN* | 0 | 0 | 0 | 0 |  | 12 | 12 | 12 | 12 | 1 | PA2495 | 13 | 13 | 13 | 13 | 0 | PA14_32380 |

**Table S7.** Antibiotic resistance genes analyzed between ST-1146 isolates and compared with *P. aeruginosa* PAO1 and *P. aeruginosa* PA14.
